# Dysbiosis of gut microbiota after cholecystectomy is associated with non‐alcoholic fatty liver disease in mice

**DOI:** 10.1002/2211-5463.13243

**Published:** 2021-07-12

**Authors:** Qihan Wang, Qifan Lu, Wentao Shao, Zhaoyan Jiang, Hai Hu

**Affiliations:** ^1^ Center of Gallbladder Disease Shanghai East Hospital School of Medicine Tongji University Shanghai China

**Keywords:** 16S rRNA sequencing, cholecystectomy, gut microbiota, NAFLD

## Abstract

Increasing evidence suggests that cholecystectomy is an independent risk factor for non‐alcoholic fatty liver disease (NAFLD). However, the underlying mechanisms that lead to hepatic lipid deposition after cholecystectomy are unclear. In this study, adult male C57BL/6J mice that underwent a cholecystectomy or sham operation were fed either a high‐fat diet (HFD) or a chow diet for 56 days. Significantly increased steatohepatitis, liver/body weight ratio, hepatic triglycerides, and glucose intolerance were observed in postcholecystectomy mice fed the HFD. Notable alterations in the composition of gut microbiota after cholecystectomy were observed in both HFD‐ and chow‐diet‐fed mice. Our results indicate that cholecystectomy alters the gut microbiota profile, which might contribute to the development of NAFLD in mice.

## Graphical abstract

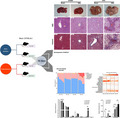

**Abbreviations**
AUC: area under the curve
FGF15: fibroblast growth factor 15
GBx: cholecystectomy
HFD: high‐fat diet
LDA: logarithmic linear discriminant analysis
LEfSe: LDA effect size
LPS: lipopolysaccharide
NAFLD: non‐alcoholic fatty liver disease
NAS: NAFLD activity score
NASH: non‐alcoholic steatohepatitis
PCoA: principal coordinate analysis
RDP: Ribosomal Database Project
SCFAs: short‐chain fatty acids

Cholecystolithiasis is a common disease and a recent epidemiological investigation showed that its incidence has exceeded 15% [1]. Laparoscopic cholecystectomy is now considered a safe and effective treatment for symptomatic cholecystolithiasis. Patients who undergo cholecystectomy account for 5% of the population [1]. However, much concern has been raised by clinicians and researchers since cholecystectomy was reported to be closely related to multiple pathological disorders such as colorectal cancer, bile reflux gastritis, and metabolic abnormalities [2].

Gallbladder removal is a risk factor of non‐alcoholic fatty liver disease (NAFLD) and increases the incidence of liver cirrhosis through the progression of chronic liver disease [3]. A strong correlation between cholecystectomy and a fatty liver has been shown by Yun *et al*. [4]. Patients with gallbladder removal have risks of increases in liver fat, serum apolipoprotein B, and insulin resistance index (HOMAIR index) 2 years after surgery [5]. A large cross‐sectional study of Asian patients showed that cholecystectomy is an independent risk factor for NAFLD [6]. The third national health and nutrition examination survey (NHANES 1988–1994) also found a significant increase in NAFLD risk after cholecystectomy [7].

NAFLD has become a major cause of chronic liver diseases and liver transplantation worldwide. The pathogenesis of NAFLD has been proposed to be the result of multiple factors such as insulin resistance, nutrient overloading, and inflammation, and associated with obesity, diabetes, cholecystolithiasis, and other metabolic syndrome‐related diseases [8]. The mechanisms by which cholecystectomy increases the risk of a fatty liver have not been elucidated.

Gut microbiota that contributes to disorders of lipid and glucose metabolisms has been a hot topic in recent years. Disorder in the composition or functioning of gut microbiota is strongly associated with various chronic metabolic diseases such as obesity, metabolic syndrome, and NAFLD [9]. In this study, we performed a cholecystectomy or sham surgery on C57BL/6 J mice and compared differences in hepatic steatosis and inflammation under a high‐fat diet challenge. We found changes in gut microbiota during the above process through large‐scale sequence analysis of 16S rDNA in cecum contents. Our results suggest that changes in gut microbiota might contribute to the development of non‐alcoholic fatty liver disease after cholecystectomy.

## Materials and methods

### Animal experiments

Twenty male C57BL/6J mice (8 weeks old) were purchased from Shanghai Model Organisms Center (Shanghai, China, license No. SCXK‐HU 2017‐0010). These specific pathogen‐free mice were housed in laminar flow cabinets of the Animal Care Facility in Shanghai East Hospital, Tongji University School of Medicine. The facility provided controlled humidity (50% ± 5%) and temperature (23 ± 2 °C) with a 12‐h light / 12‐h dark cycle. When the mice had adapted to the environment, they were randomly and equally assigned to undergo a cholecystectomy or sham operation.

Cholecystectomy mice (GBx) were subjected to a substernal median incision, and the gallbladder was removed after gallbladder duct ligation. Sham mice were subjected to a substernal median incision, and the abdominal wall incision was closed immediately after gallbladder inspection. The abdominal wall incision was sutured and disinfected with 75% alcohol 3 days after the operation.

Five cholecystectomy mice and five sham mice were fed a high‐fat diet (4.5 kcal·g^−1^, calories comprised 40% fat, 20% protein, 40% carbohydrate, and 0.2% cholesterol) and high sugar water. The other five cholecystectomy mice and five sham mice were fed a chow diet (2 kcal·g^−1^, calories comprised 10% fat, 20% protein, 70% carbohydrate, and 0.02% cholesterol) and pure water.

During the 56‐day experiment, all the mice consumed the designated water and food ad libitum. On the day of sacrifice, the mice were euthanized by exsanguination after i.p. injection of chloral hydrate (350 mg·kg^−1^ body weight). All animal experiments were reviewed and approved by the Animal Care Committee at Shanghai East Hospital, Tongji University School of Medicine (approval no. TJLAC‐019‐048).

### Histological analysis

Formalin‐fixed and paraffin‐embedded liver tissues were stained with hematoxylin and eosin and Masson’s trichrome. Two investigators who were blinded to the treatments independently evaluated the sections and assigned a NAFLD activity score (NAS) using the non‐alcoholic steatohepatitis (NASH) Clinical Research Network Scoring System [10]. The NAS is the unweighted sum of three semiquantitative features: steatosis (0–3), lobular inflammation (0–2), and hepatocellular ballooning (0–2) scores. NAS of > 5 correlates with a diagnosis of NASH and biopsies with scores of < 3 are diagnosed as ‘not NASH’. A fibrosis score (0–4) was determined by assessing the area stained with Masson’s trichrome in the liver specimens.

### Quantitative real‐time PCR analysis

Total RNA was isolated from liver and ileum tissues using TRIZOL (Life Technologies, Carlsbad, CA, USA) following the standard protocol. First‐strand cDNA was synthesized using a Total RNA with High‐Capacity cDNA Reverse Transcription Kit (Applied Biosystems, Foster City, CA, USA) in accordance with the manufacturer's instructions. The original amount of specific transcripts was detected by real‐time PCR with PowerUp SYBR Green Master Mix (Applied Biosystems, Austin, TX, USA). Each sample was evaluated in triplicate. Normalization of relative expression was calculated by the comparative Ct (2^−ΔΔCt^) method with GAPDH gene expression. Primers are listed in Table 1.

**Table 1:** Primer sequences for real‐time RT‐PCR

Gene	Forward (5′‐3′)	Reverse (5′‐3′)
ABCB11	CAATAGACAGGCAACCCGTCA	GTGGAACTCAATTTCGCCCTT
CYP7A1	AGCAACTAAACAACCTGCCAGTACTA	GTCCGGATATTCAAGGATGCA
IL‐10	GCTCTTACTGACTGGCATGAG	CGCAGCTCTAGGAGCATGTG
IL‐1β	TTGAAGAAGAGCCCATCCTC	CAGCTCATATGGGTCCGAC
TNF‐α	TAGCCAGGAGGGAGAACAGA	TTTTCTGGAGGGAGATGTGG
FGF15	GGTCGCTCTGAAGACGATTG	CGCGCTCATGCAGAGGTA
Occludin	ATGTCCGGCCGATGCTCTC	TTTGGCTGCTCTTGGGTCTGTAT
ZO‐1	ACCCGAAACTGATGCTGTGGATAG	AAATGGCCGGGCAGAACTTGTGTA

### Biochemical analyses

Hepatic total triglycerides and total cholesterol were detected by enzymatic colorimetric assays using a biochemical auto‐analyzer (Roche Diagnostics GmbH, Mannheim, Germany) and commercial enzymatic test kits (triglycerides, GPO‐PAP, Cat. No. 11730711; cholesterol CHOD‐PAP, Cat. No. 11875540, Roche Diagnostics GmbH, Mannheim, Germany).

### Illumina library generation and sequencing

High‐quality total microbial DNA was isolated from cecum content samples using an E.Z.N.A. Stool DNA Kit (Omega Bio‐Tek, Norcross, GA, USA). The V4 and V5 regions of the 16S ribosomal RNA gene were amplified by PCR using a forward primer (515F: 5′‐barcode‐GTG CCA GCM GCC GCG G‐3′) and reverse primer (907R: 5′‐CCG TCA ATT CMT TTR AGT TT‐3′). The barcode in the forward primer was an eight‐base sequence unique to each sample for sorting. Amplicons were purified using an axyprep DNA gel extraction kit (Axygen Biosciences, Union City, CA, USA), quantified, and then pooled in equimolar amounts. In accordance with standard protocols, purified amplicons were applied to an Illumina miseq platform for 250‐nucleotide paired‐end read assembly.

### Bioinformatics analyses

Using QIIME software, we demultiplexed, quality filtered, and analyzed raw Illumina fasta files. All 16S rRNA gene sequences were analyzed and assigned to a taxonomical hierarchy, which indicated their phylogenetic affiliation, by the Ribosomal Database Project (RDP) Classifier (version 11.1, http://rdp.cme.msu.edu/) using a confidence threshold of 70%. The composition of gut microbiota was analyzed and compared between groups by computing the relative abundances of the various phyla and genera from each sample. Weighted and unweighted unifrac distances between samples were calculated to compress dimensionality and obtain two‐dimensional principal coordinate analysis (PCoA) plots. Online LDA effect size (LEfSe) analysis was performed in accordance with logarithmic linear discriminant analysis (LDA) scores (http://huttenhower.sph.harvard.edu/galaxy/). The threshold of the LDA score for discriminative features was < 2.

### Statistical analyses

Data are expressed as means ± SEM. Differences between multiple groups were determined by one‐way ANOVA and Tukey’s multiple comparisons test. A value of *P* < 0.01 indicates statistically significant differences. All statistical tests were performed by Prism 9 (GraphPad Software, San Diego, CA, USA).

## Results

### GBx + HFD aggravates metabolic disorders compared with Sham HFD mice

**Fig. 1:**
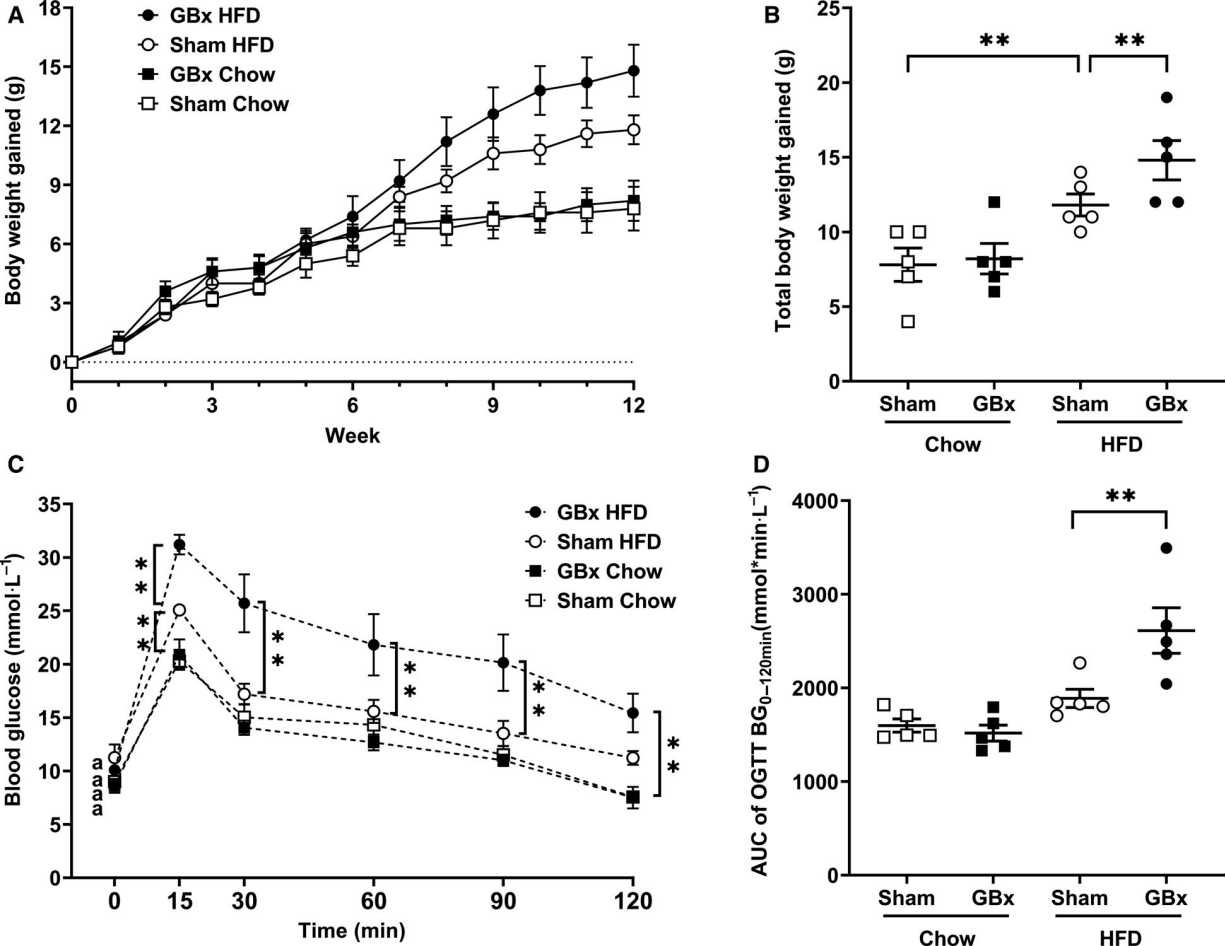
GBx + HFD aggravates metabolic disorders compared with sham HFD mice. (A and B) Body weight gains. (C and D) OGTT and AUC for the OGTT (BG 0–120 min). The error bars indicate the SEM; *n* = 5 per group; **P < 0.01 by one‐way ANOVA and Tukey’s multiple comparisons test. OGTT, oral glucose tolerance test; AUC, area under the curve.

HFD caused a significant increase in weight gain in both GBx and sham mice (*P* < 0.01). GBx HFD mice gained more weight than sham HFD mice (*P* < 0.01) (Fig. 1A,B). GBx did not affect body weight with the chow diet. GBx HFD mice showed a distinctive change in the blood glucose curve that had a significantly higher peak, slower rate of decline, and noticeably increased area under the curve (AUC; *P* < 0.01; Fig. 1C,D).

**Fig. 2:**
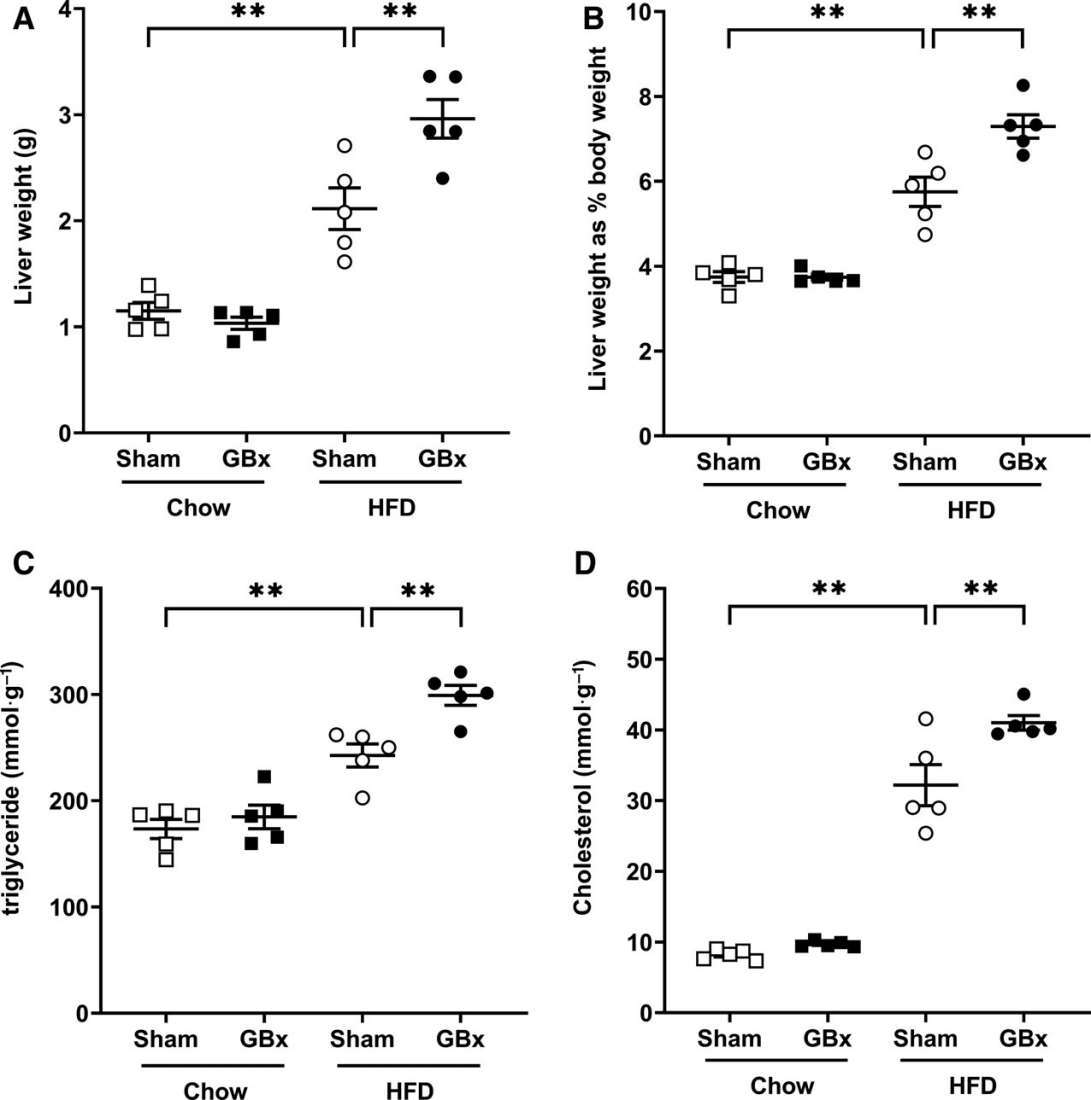
GBx + HFD increases the weight and lipid content of the liver. (A) Liver weight. (B) Weight ratio of the liver/body. (C) Liver triglyceride content. (D) Liver cholesterol content. The error bars indicate the SEM; *n* = 5 per group; **P < 0.01 by one‐way ANOVA and Tukey’s multiple comparisons test.

HFD led to significant increases in the liver weight and liver/body weight ratio compared with the chow diet. More serious elevation was observed in GBx HFD mice (Fig. 2A,B). Hepatic lipid tests showed that GBx further increased hepatic cholesterol and triglyceride contents in HFD mice (Fig. 2C,D).

Collectively, these results suggest that cholecystectomy aggravated metabolic disorders, such as obesity, increased liver lipid content, and impaired glucose tolerance in HFD feeding mice.

### GBx causes more severe fatty liver, steatohepatitis, and fibrosis in mice fed an HFD

**Fig. 3:**
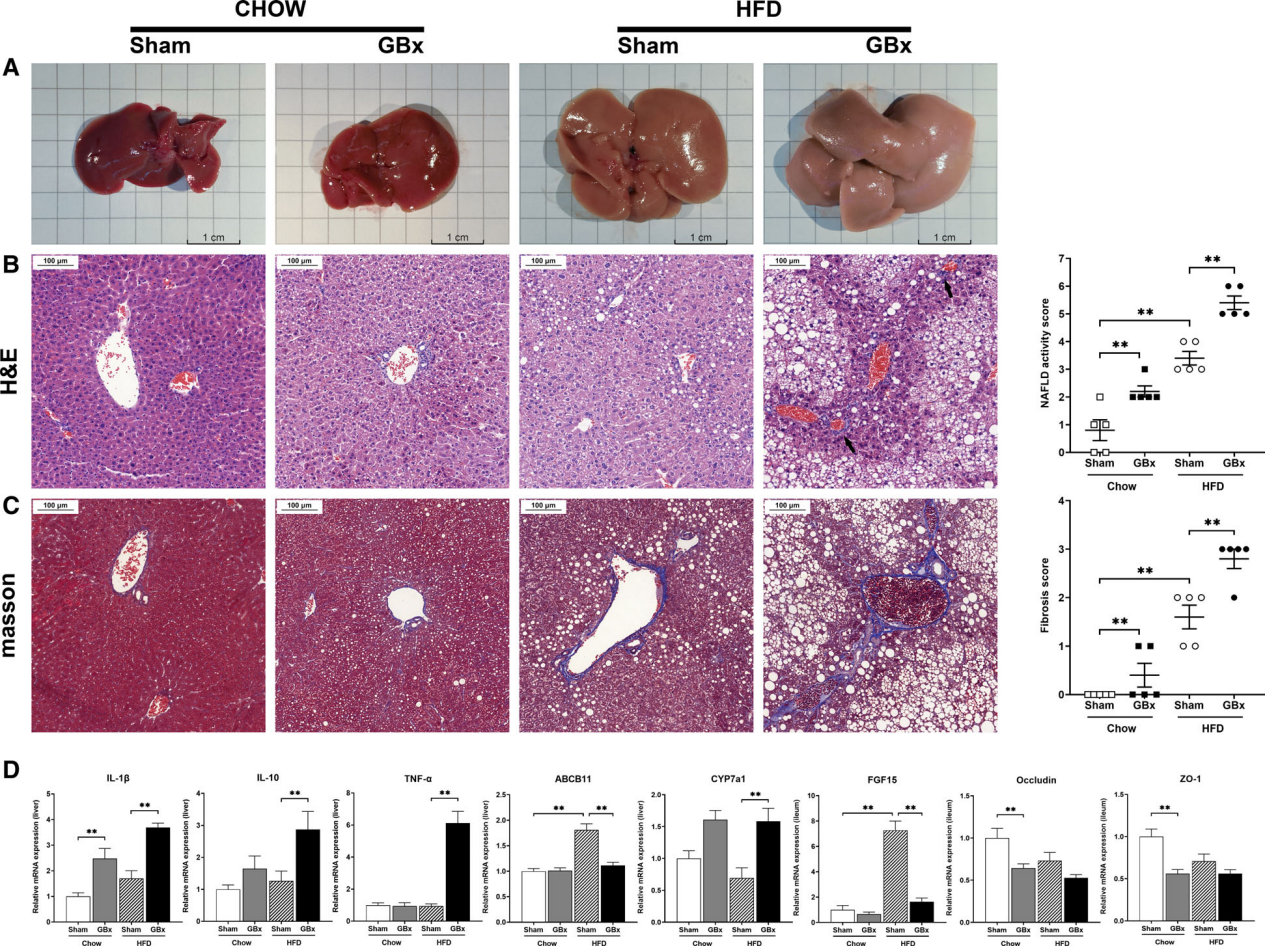
GBx causes a severe fatty liver, steatohepatitis, and fibrosis in mice fed an HFD. (A) Representative gross morphology of the liver. Scale bar = 1 cm. (B) Representative H&E staining of liver sections. Scale bar = 100 μm. NAFLD activity scores are shown. Arrows indicate immune cell accumulation. (C) Representative Masson’s trichrome staining of liver sections. Scale bar = 100 μm. Fibrosis scores are shown. (D) Relative mRNA levels of IL‐1β, IL‐10, TNFα, ABCB11, and CYP7a1 in liver tissues and those of FGF15, occludin, ZO‐1 in ileum tissues. The error bars indicate the SEM; *n* = 5 per group; **P < 0.01 by one‐way ANOVA and Tukey’s multiple comparisons test.

HFD resulted in significant changes in the appearance of livers. GBx HFD mice showed a more similar appearance to a NAFLD liver than sham HFD mice (Fig. 3A). Further examination of liver sections revealed steatohepatitis characterized by steatosis and lobular inflammation in GBx HFD mice, whereas only steatosis with mild inflammation was observed in sham HFD mice (Fig. 3B). HFD feeding resulted in mild collagen deposition in the liver tissue of sham HFD mice. GBx HFD mice presented severe fibrotic injury with significantly more collagen‐stained areas (Fig. 3C). NASs and fibrosis scores confirmed the increased severity of liver histology in HFD mice after GBx.

### GBx affects liver inflammation, bile acid metabolism, and tight junctions of the intestinal epithelium

In liver tissue, the relative mRNA expression of proinflammatory cytokines IL‐1β and TNF‐α was significantly higher in GBx HFD mice than in other groups (*P* < 0.01). There was also a higher relative expression of the anti‐inflammatory cytokine IL‐10 in GBx HFD mice than that in other groups (*P* < 0.01). ABCB11 was significantly increased in sham HFD mice and decreased in GBx HFD mice (*P* < 0.01). The mRNA expression level of CYP7a1 was remarkably reduced in sham HFD mice and higher in GBx HFD mice (*P* < 0.01). In ileum tissues, the relative mRNA expression of FGF15 was significantly higher in sham HFD mice than in other groups (*P* < 0.01). The mRNA expression of tight junction‐related proteins occludin and ZO‐1 was reduced largely in both GBx chow and GBx HFD mice (*P* < 0.01; Fig. 3D).

### Alteration of gut microbiota after GBx

**Fig. 4:**
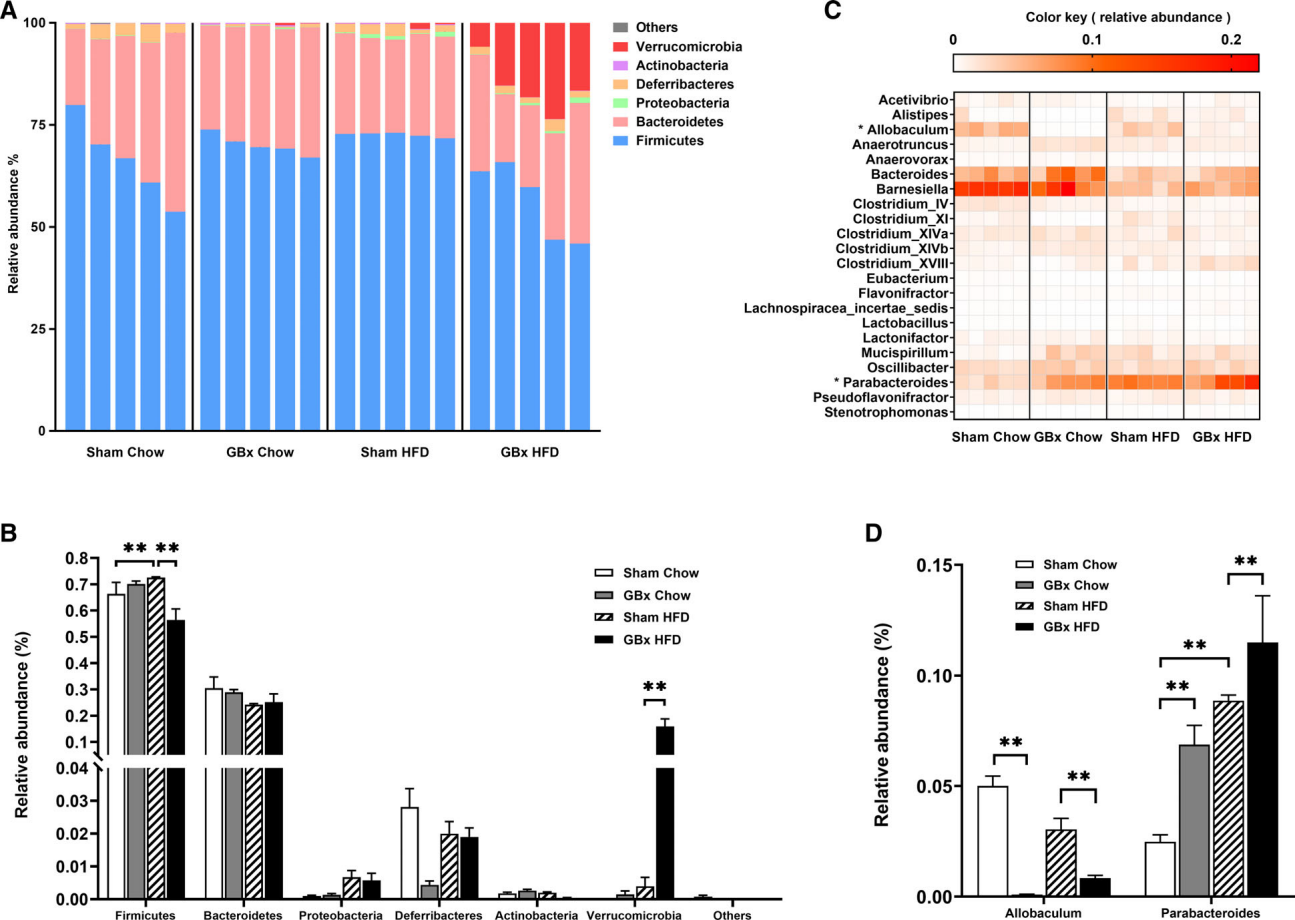
Alteration of gut microbiota after GBx. (A) Gut microbiota composition in each mouse at the phylum level. (B) Gut microbiota composition at the phylum level summarized by groups. (C) Heat map of gut microbiota at the genus level. The 22 core genera shared by all samples tested are displayed and unclassified subgroups are excluded. (D) Relative abundances of Allobaculum and Parabacteroides. The error bars indicate the SEM; *n* = 5 per group; **P < 0.01 by one‐way ANOVA and Tukey’s multiple comparisons test.

Through phyla‐level analysis (Fig. 4A,B), we found that *Firmicutes* was the most dominant gut microbiota phylum; this comprised 66.3% of gut microbiota in the sham chow group and 70.1% in the GBx chow group. *Firmicutes* increased to 72.6% in sham HFD mice (*P* < 0.01 vs sham chow) but was significantly reduced to 56.4% in the GBx HFD group (*P* < 0.01 vs sham HFD). Conversely, the relative abundances of *Verrucomicrobia* were < 0.2% in GBx chow, sham chow, and sham HFD mice, but it was significantly increased to 15.9% in GBx HFD mice (*P* < 0.01).

A heat map was constructed of the most abundant core genera that were shared by all tested samples (Fig. 4C). An obvious decrease in the genus *Allobaculum* in GBx mice compared with sham mice was observed. Conversely, the genus *Parabacteroides* was increased (Fig. 4D).

**Fig. 5:**
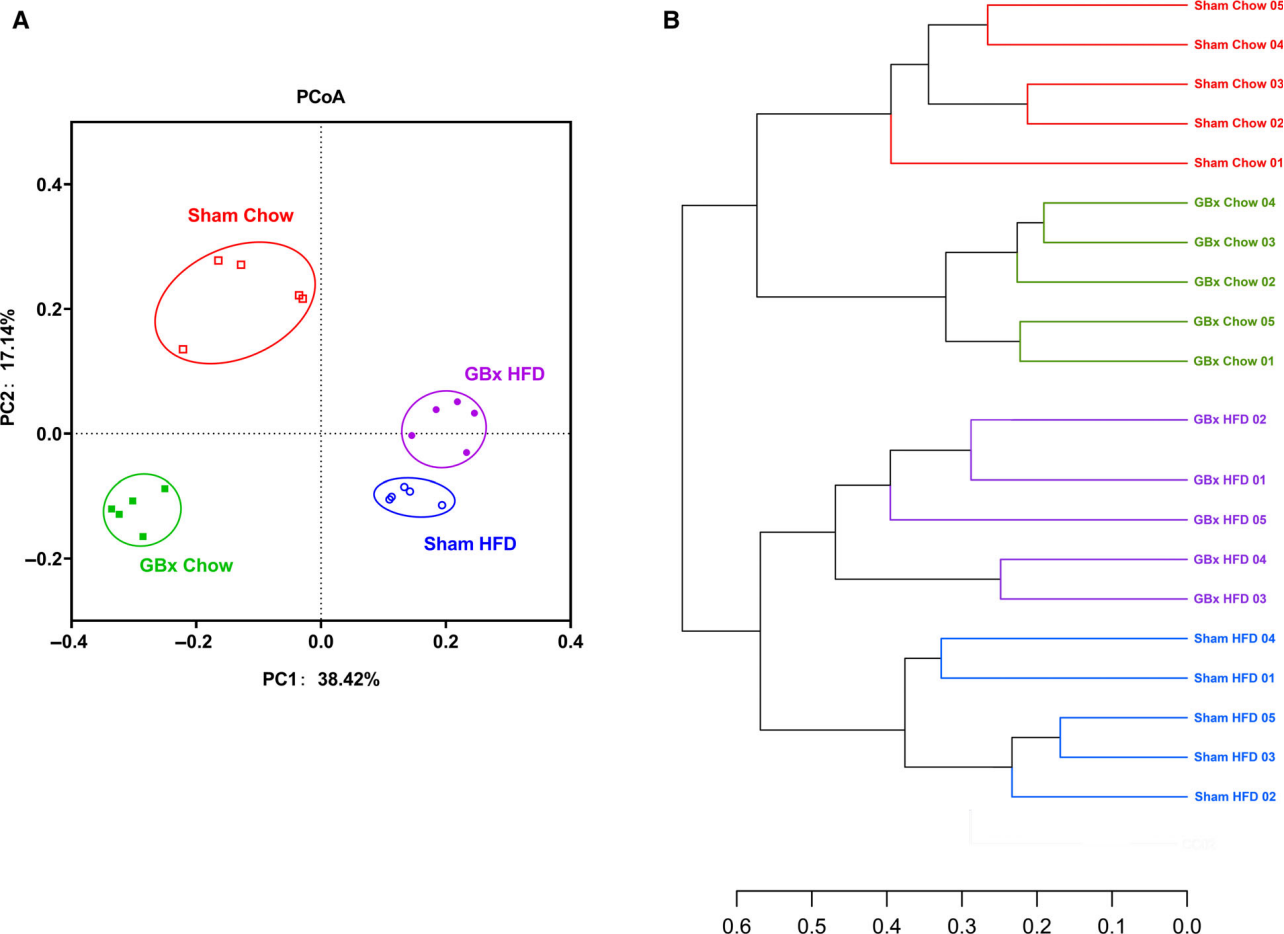
β‐diversity and phylogenetic analysis of gut microbiota. (A) Principal coordinates analysis (PCoA, weighted). (B) Hierarchical cluster tree of phylogenetic analysis.

Principal coordinates analysis (PCoA) is a nonconstrained data dimensionality reduction analysis method that is used to assess the similarity or difference of sample community composition. This method determines potential factors that influence the difference in sample community composition through dimensionality reduction. Figure 5A shows significant divergences in the composition of gut microbiota among the groups. This result revealed that, similar to HFD, GBx exerted a significant influence on gut microbiota.

A hierarchical cluster tree is a multivariate statistical analysis method that classifies samples in accordance with their degree of affinity in species composition. The similarity and difference in sample composition are defined by the structure of branches. The closer the branches are, the more similar the species composition of the two samples is. Similar to the conclusion of PCoA, cluster analysis showed significant differences between any two groups (Fig. 5B) and revealed a significant influence caused by GBx on gut microbiota.

**Fig. 6:**
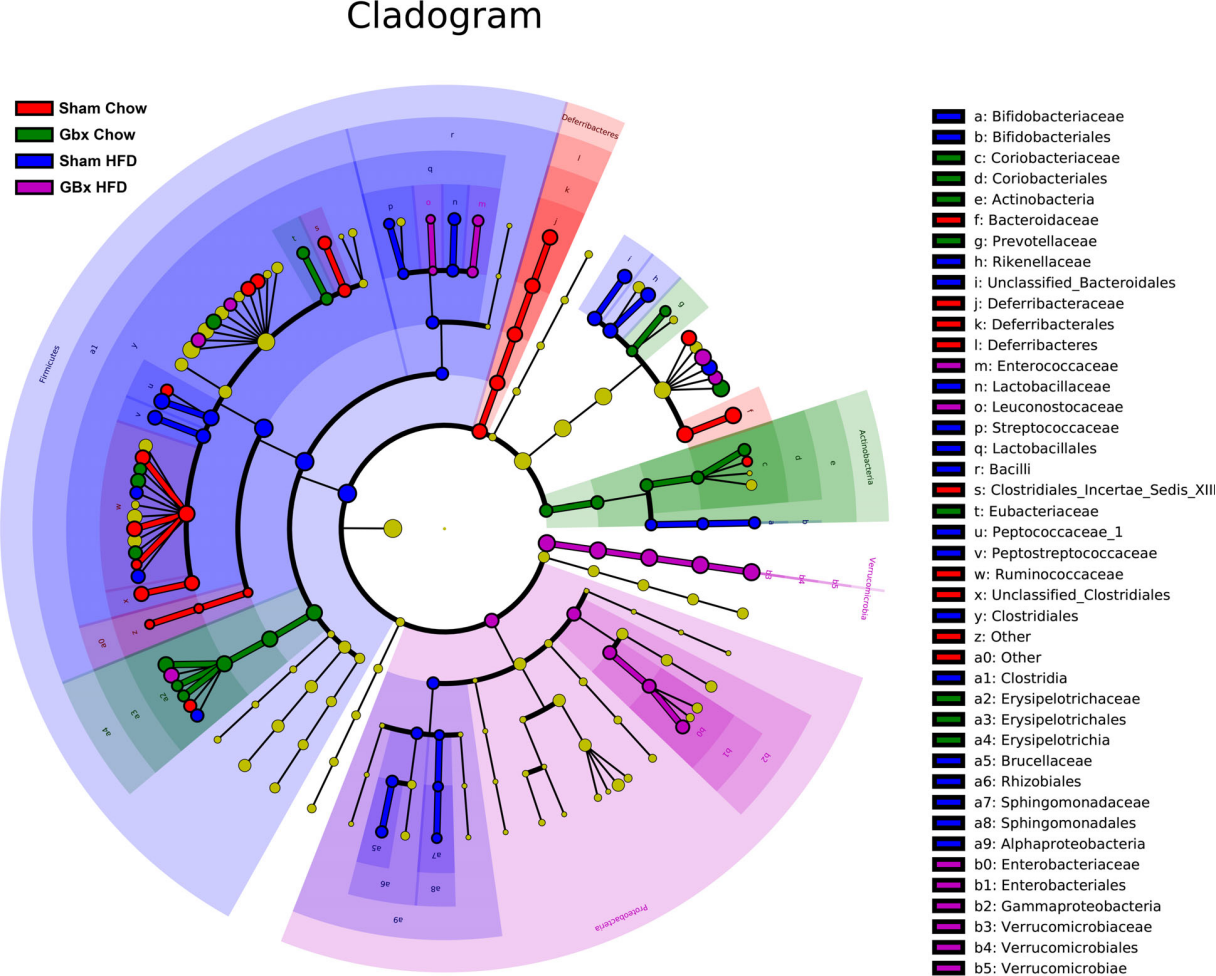
Cladogram of taxonomic analysis. The diameter of each circle is proportional to the taxa abundance.

A cladogram generated from LEfSe analysis identified the communities or species with a significant effect on the difference in sample classification at different levels. As expected, GBx HFD mice showed a significant decrease in *Firmicutes* and a remarkable higher abundance of *Verrucomicrobia* compared with the other groups (Fig. 6).

## Discussion

In this study, C57BL/6J mice that underwent sham surgery and were fed the HFD for 56 days developed obesity and mild fatty liver disease. Severe non‐alcoholic steatohepatitis (NASH), significant increases in the liver/body weight ratio and hepatic triglycerides, and glucose intolerance were observed in postcholecystectomy mice fed the HFD. A study by Amigo *et al*. [11] confirmed increased serum triglycerides and very low‐density lipoprotein content in cholecystectomy mice. This is related to increased intake of free fatty acids from peripheral adipose tissue through blood circulation by the liver, which leads to the accumulation of lipids in the liver. The findings of our study provided evidence indicating that cholecystectomy promotes the development of NAFLD induced by HFD.

Germ‐free mice lacking gut microbiota show resistance to liver steatosis induced by an HFD [12], and this resistance disappears after fecal transplantation [13]. Hepatic steatosis can be alleviated by treatment with antibiotics or probiotics [14, 15]. Many cross‐sectional clinical studies have confirmed significant differences in the gut microbiota of NAFLD patients compared with normal controls [16, 17]. Because the pathogenesis of NAFLD is closely related to disturbance of gut microbiota, we explored the relationship between the distribution of gut microbiota and the development of a fatty liver in mice after cholecystectomy. The distribution of gut microbiota in mice after cholecystectomy was significantly altered compared with the sham group. During HFD feeding to sham‐operated mice to induce obesity and a mild fatty liver, *Firmicutes* as the dominant gut microbiota at the phylum level had a significant increase in abundance. This is consistent with previous studies on the effect of high‐calorie diets on gut microbiota [18, 19]. However, as the disease progresses from mild/moderate NAFLD to advanced NASH and fibrosis in HFD mice after cholecystectomy, the *Firmicutes* phylum had a statistically significant decrease in abundance, while the *Verrucomicrobia* phylum increased. Several metagenomics‐based studies on the distribution of gut microbiota in adult and child patients with NASH have also reached similar conclusions [16, 20, 21].

Compared with sham HFD mice, the mRNA expression levels of CYP7a1, ABCB11, and FGF15 in GBx HFD mice were significantly different. The CYP7a1 gene encodes the rate‐limiting enzyme in the classic bile acid synthesis pathway of the liver. The ABCB11 gene encodes the bile salt output pump protein, which is mainly located in the bile duct membrane of hepatocytes and is the major canalicular bile salt export pump. Bile acids act on the nuclear receptor FXR in ileal enterocytes to induce the expression of mouse fibroblast growth factor 15 (FGF15). FGF15 is secreted into enterohepatic circulation and downregulates CYP7a1 expression in the liver to limit bile acid synthesis [22, 23]. The gallbladder is a remote organ outside the intestines, where bile is stored and concentrated, and regulates gut microbiota homeostasis. Contraction and emptying of the gallbladder play an important role in enterohepatic circulation of bile acid. After cholecystectomy, bile acids are continuously secreted into the intestines with bile and the rhythm of bile acids that enter the digestive tract after meals disappears. The enterohepatic circulation rate of bile acids accelerates, and the amount of excrement loss also increases. Bile acids as potent inhibitors of bacterial growth exert selective pressure on gut microbiota and regulate the abundance and composition of gut microbiota [24]. Disrupting the interaction between bile acids and gut microbiota promotes inflammation and digestive tract diseases [25]. Therefore, changes in bile acid metabolism after cholecystectomy may be a major factor in the disturbance of the gut microbiota in mice.

The mRNA expression of tight junction‐related proteins occludin and ZO‐1 in ileum tissues of GBx mice was decreased significantly. Tight junctions are one of the most important physiological and pathological regulators of intestinal permeability. Current evidence suggests that intestinal permeability disruption promotes the onset of NAFLD [26, 27]. The tight junction proteins block paracellular gaps between intestinal epithelial cells and prevent harmful compounds and microbes from passing through [28]. Once the tight junction is disrupted, excessive non‐self‐antigens cross the intestinal mucosa and enter the portal vein bloodstream, which triggers subsequent pathological reactions in the liver and eventually leads to NASH [29]. Disruption of the intestinal permeability makes flora products, such as lipopolysaccharide (LPS), enter the bloodstream largely through the intestinal wall and enter the liver via the portal vein. Endotoxemia induced by endotoxin translocation activates Toll‐like receptor 4 in the liver to further activate downstream transcription factors, which triggers an immune response and liver inflammation [30]. The association of increased intestinal permeability is stronger in NASH patients, which demonstrates that the inflammatory changes observed in NASH might be caused by increased intestinal permeability [31]. Therefore, the increased expression of inflammatory cytokines and severe steatohepatitis observed in cholecystectomy mice might be associated with disruption of intestinal tight junctions and increased intestinal permeability.

In our study, we observed a decrease in *Allobaculum* and a higher abundance of *Parabacteroides* caused by cholecystectomy. *Allobaculum* and *Parabacteroides* are involved in the fermentation of short‐chain fatty acids (SCFAs) in the intestines [32, 33]. SCFAs are mainly produced by microbial anaerobic fermentation in the intestinal tract and mainly comprise acetic acid, propionic acid, and butyric acid [34]. As a nutrient of intestinal epithelial cells, SCFAs not only meet most of their energy requirements but also act on white blood cells and endothelial cells to regulate the production of cytokines, arachidonic acid, and chemokines, and guide the differentiation of T cells to play a role in the immune response of intestinal and peripheral tissues [35, 36]. In a mouse model of high‐fat diet–mediated NAFLD, short‐chain fatty acid supplementation improved liver steatosis [37]. HFD lowers the abundance of *Allobaculum* [38]. Effective clinical drugs, such as berberine and metformin [39], and food interventions, such as probiotics [40] and grape extracts [41], that help improve metabolism have been shown to increase the presence of *Allobaculum*. *Allobaculum* significantly correlates with resistance against NAFLD development by improving intestinal integrity and increasing Reg3γ levels in the colon [15]. *Parabacteroides*, a bile‐resistant anaerobic Gram‐negative bacterium, is significantly higher in the gut microbiota of NAFLD patients compared with normal controls. Henning *et al*. [42] have shown that the relative abundance of *Parabacteroides* is associated with fat accumulation.

In summary, our study showed that cholecystectomy aggravated metabolic disorder and steatohepatitis induced by HFD, which were accompanied by a dramatically altered composition of gut microbiota. These results suggest that gut microbiota disorder caused by cholecystectomy might contribute to the development of NAFLD. We propose the following hypothesis: Cholecystectomy alters normal bile acid metabolism, which leads to gut microbiota disorder, changes the composition and content of microbial metabolites, affects the intestinal internal environment and intestinal wall permeability, further leads to a severe inflammatory reaction in the liver, and eventually causes NAFLD induced by a high‐fat diet to develop into NASH.

## Conflict of interest

The authors declare no conflict of interest.

## Author contributions

QHW performed the experiments, analyzed the data, and wrote the manuscript. QFL and WTS participated in the data collection. HH and ZYJ participated in conception and oversight of the study, supervision, data analysis, and manuscript editing.

## Data Availability

Data sets used or analyzed during this study are available from the corresponding author upon reasonable request.
